# Assessment of Risk Factors and Outcomes of Severe Ehrlichiosis Infection

**DOI:** 10.1001/jamanetworkopen.2020.25577

**Published:** 2020-11-17

**Authors:** Kevin Kuriakose, April C. Pettit, Jonathan Schmitz, Abelardo Moncayo, Karen C. Bloch

**Affiliations:** 1 Section of Infectious Disease, Renown Health, Reno, Nevada; 2 Department of Medicine, School of Medicine, University of Nevada, Reno; 3 Division of Infectious Diseases, Department of Medicine, Vanderbilt University Medical Center, Nashville, Tennessee; 4 Department of Pathology, Microbiology, and Immunology, Vanderbilt University Medical Center, Nashville, Tennessee; 5 Vector-Borne Disease Section, Tennessee Department of Health, Nashville; 6 Department of Health Policy, Vanderbilt University Medical Center, Nashville, Tennessee

## Abstract

**Importance:**

Ehrlichiosis cases in the US have increased more than 8-fold since 2000. Up to 57% of patients with ehrlichiosis require hospitalization and 11% develop a life-threatening complication; however, risk factors for serious disease are not well documented.

**Objective:**

To examine risk factors associated with severe ehrlichiosis.

**Design, Setting, and Participants:**

An analytic cross-sectional study of patients diagnosed with ehrlichiosis by polymerase chain reaction (PCR) between January 1, 2007, and December 31, 2017, was conducted in a single tertiary-care center in a region endemic for ehrlichiosis. Analysis was performed from February 27, 2018, to September 9, 2020. A total of 407 positive *Ehrlichia* PCR results were identified from 383 unique patients, with 155 unique patients meeting study criteria. Patients hospitalized at other institutions who had a positive *Ehrlichia* PCR performed as a reference test (n = 222) were excluded as no clinical data were available. Electronic medical record review was performed to collect demographic, clinical, laboratory, treatment, and outcomes data. Cases were excluded when there were insufficient clinical data to assess the severity of illness (n = 3) and when the clinical illness did not meet the case definition for ehrlichiosis (n = 3).

**Exposures:**

Date of presentation, onset of symptoms, date of PCR testing, date of treatment initiation, site of care, age, birth sex, race/ethnicity, Charlson Comorbidity Index, trimethoprim with sulfamethoxazole use within the prior 2 weeks, and immunosuppression.

**Main Outcomes and Measures:**

Requirement for intensive care unit (ICU) admission.

**Results:**

Of the 155 patients who met inclusion criteria, 99 patients (63.9%) were men, and 145 patients (93.5%) identified as non-Hispanic White; median age was 50 years (interquartile range, 23-64 years). Intensive care unit admission was indicated in 43 patients (27.7%), 94 patients (60.6%) were hospitalized on general medical floors, and 18 patients (11.6%) received care as outpatients. In adjusted analysis, time to treatment initiation was independently associated with an increased risk for ICU admission (adjusted prevalence ratio [aPR], 1.09; 95% CI, 1.04-1.14; *P* < .001). Documentation of tick exposure was independently associated with a decreased risk for ICU admission (aPR, 0.54; 95% CI, 0.34-0.86; *P* = .01). There appeared to be a nonsignificant change toward a decreased need for ICU care among immunosuppressed persons (aPR, 0.51; 95% CI, 0.26-1.00; *P* = .05).

**Conclusions and Relevance:**

This study suggests that delay in initiation of doxycycline therapy is a significant factor associated with severe ehrlichiosis. Increased recognition of infection by front-line clinicians to promote early treatment may improve outcomes associated with this increasingly common and life-threatening infection.

## Key Points

**Question:**

What risk factors are associated with severe ehrlichiosis?

**Findings:**

This cross-sectional study including 155 patients identified a delay in doxycycline therapy as a significant factor associated with severe ehrlichiosis. Documentation of tick exposure was independently associated with a decreased need for intensive care unit admission, and a change toward a decreased need for intensive care unit admission among immunosuppressed persons was identified.

**Meaning:**

In this study, delay in initiation of empirical doxycycline therapy appears to be a risk factor for severe ehrlichiosis; education focused on early recognition and treatment may decrease morbidity associated with this infection.

## Teaser

This cross-sectional study examines risk factors associated with severity of infection in patients with ehrlichiosis.

## Introduction

Ehrlichiosis, a tick-borne infection caused by 3 closely related species (*Ehrlichia chaffeensis, E ewingii*, and *E muris eauclairensis*), is an increasingly recognized cause of human infection. The number of cases reported to the US Centers for Disease Control and Prevention (CDC) has increased more than 8-fold since reporting began in 2000.^1^ In 2017, 1642 cases of *E chaffeensis* were identified in the US, representing a 19% increase compared with the previous year.^1^ The 2 other species are less commonly diagnosed, with a total of 218 cases of *E ewingii* and 115 cases of *E muris eauclairensis* reported since dedicated surveillance began.^1^ However, reported cases of tick-borne infections almost certainly underestimate the actual burden of disease owing to limitations in laboratory confirmation and passive surveillance.^2^ For instance, it is estimated that the number of cases of Lyme disease reported to the CDC annually represents a 10-fold underrepresentation of the true incidence.^3^

Clinical manifestations of ehrlichiosis are nonspecific.^4^ Definitive diagnosis requires laboratory confirmation, defined as a 4-fold increase in IgG antibody titer between paired serum samples or detection of *E chaffeensis* or *E ewingii* DNA by polymerase chain reaction (PCR) in a clinical specimen.^5^ Serologic diagnosis is limited by delayed seroconversion^6^ and paired testing is inconsistently performed.^7^ Polymerase chain reaction has been shown to be highly sensitive during acute illness.^8^

Despite the availability of effective antibiotic therapy, life-threatening *Ehrlichia* infections are common. Among probable and confirmed *E chaffeensis* cases reported to the CDC between 2008 and 2012, 57% required hospitalization and 11% had life-threatening complications.^7^ The cumulative case fatality rate was 1% overall. Among the subset of cases with confirmed laboratory infection,^5^ the overall case fatality rate was 2%, but increased to 14% in children younger than 5 years and 53% in those older than 70 years.^7^ Previous reports have identified immunocompromised state, including HIV infection and solid organ transplant, as well as recent treatment with trimethoprim plus sulfamethoxazole, as risk factors for serious infections.^7,9,10,11,12,13,14,15,16,17,18,19,20,21,22^ More recent studies using PCR for diagnosis of ehrlichiosis have not substantiated this association.^23,24^

Identification of populations at increased risk for serious ehrlichiosis is crucial to improving prevention and treatment strategies. To examine risk factors for severe ehrlichiosis, we identified patients at a tertiary care center with a positive *Ehrlichia* PCR over an 11-year period and elucidated characteristics associated with need for intensive care unit (ICU) admission.

## Methods

### Study Population

We conducted an analytic cross-sectional study to capture unique patients with ehrlichiosis identified by a positive PCR result on clinical specimens submitted for testing to the Molecular Infectious Diseases Laboratory at Vanderbilt University Medical Center between January 1, 2007, and December 31, 2017. This laboratory-developed test amplifies a segment of the ehrlichial 16S ribosomal RNA gene, with probe-based detection of amplicons by enzyme immunoassay.^25^ The primers of the clinical use assay at Vanderbilt University Medical Center broadly target *E chaffeensis*, *E ewingii*, and *Anaplasma phagocytophilum* with an analytic sensitivity of approximately 50 DNA copies/mL in whole-blood matrix for *Ehrlichia* and approximately 100 copies/mL for *Anaplasma*, but positive results do not differentiate between the species. Vanderbilt University Medical Center Institutional Review Board approval was obtained for all aspects of this study with a waiver of informed consent. Data were deidentified and entered into an encrypted Excel (Microsoft Corp) worksheet. This study followed the Strengthening the Reporting of Observational Studies in Epidemiology (STROBE) reporting guideline for cross-sectional studies.

Patients with a positive PCR result underwent review of their electronic medical records and those who did not have a clinically compatible illness were excluded. In cases with multiple positive PCR results, the initial test was used for the date of diagnosis. Patients hospitalized or cared for by a clinician at an external institution or who had insufficient information to ascertain severity of illness were excluded.

### Data Collection and Study Definitions

An electronic medical records review was performed to collect demographic, clinical, laboratory, treatment, and outcomes data. Demographic characteristics included age at presentation, birth sex, and self-reported race/ethnicity. Tick exposure was ascertained through documentation of self-report by patients or family members. Clinical characteristics included Charlson Comorbidity Index level,^26^ trimethoprim plus sulfamethoxazole use within the previous 2 weeks, and immunosuppression.

Immunosuppression was defined as the presence of one of the following factors in the 3 months preceding diagnosis: HIV infection, neutropenia (neutrophil count ≤0.5 × 10^3^/μL [to convert to ×10^9^/L, multiply by 1]), end-stage kidney disease requiring chronic dialysis, solid organ or bone marrow transplantation, or use of immunosuppressing medications, defined as corticosteroids administered as 20 mg/d or more of oral prednisone or equivalent, tumor necrosis factor-α inhibitors, anti-CD20 monoclonal antibodies, systemic chemotherapy, or other immunosuppressant medications, such as azathioprine and methotrexate.

Clinical variables included presence of rash, fatigue, diarrhea, abdominal pain, nausea/vomiting, cough, dyspnea, myalgias, arthralgias, headache, meningismus, seizures, and altered mental status. Laboratory data included white blood cell count, platelet count, sodium level, aspartate aminotransferase level, alanine aminotransferase level, alkaline phosphatase level, total bilirubin level, and cerebrospinal fluid white blood cell count. When sequential results were available, the most abnormal value (nadir or apex) was included. Treatment data included day of symptom onset, day of initial health care contact, day of positive PCR result, and day of doxycycline initiation.

The primary exposure of interest was immunosuppression as defined above, and the primary outcome of interest was admission to an ICU at any time during the illness. This outcome was ascertained via electronic medical record review and a recorded ICU stay of any duration. Those not requiring ICU admission included patients whose treatment was managed in the outpatient setting or in non-ICU hospital wards.

### Statistical Analysis

Data analysis was conducted from February 27, 2018, to September 9, 2020. Categorical variables were determined with frequencies and proportions and compared using the Fisher exact test. Continuous variables were measured using medians and interquartile ranges (IQRs) and compared using the Wilcoxon rank sum test. Modified Poisson regression with robust variance was used to estimate the adjusted prevalence ratio (aPR) and 95% CI of ICU care.^27^ Variables were chosen for the multivariable analysis a priori; all demographic characteristics (age, birth sex, race/ethnicity), factors associated with severity of disease in previous studies (immunosuppression and trimethoprim plus sulfamethoxazole use), clinical characteristic variables that would not independently favor transfer to the ICU with *P* values ≤.05 in the unadjusted analyses (tick exposure and rash), and time from initial health care contact to treatment initiation were included. Age was modeled with restricted cubic splines to relax linear distribution assumptions and to allow for model flexibility. All *P* values were 2-sided and considered statistically significant at <.05. Analyses were conducted using Stata, version 14.2 (StataCorp). Case fatality ratio was calculated as the proportion of patients who died over the total number of patients. A violin plot was generated to visualize distribution of days from first health care contact to treatment initiation.^28^

## Results

### Demographic Characteristics

**Figure 1.**
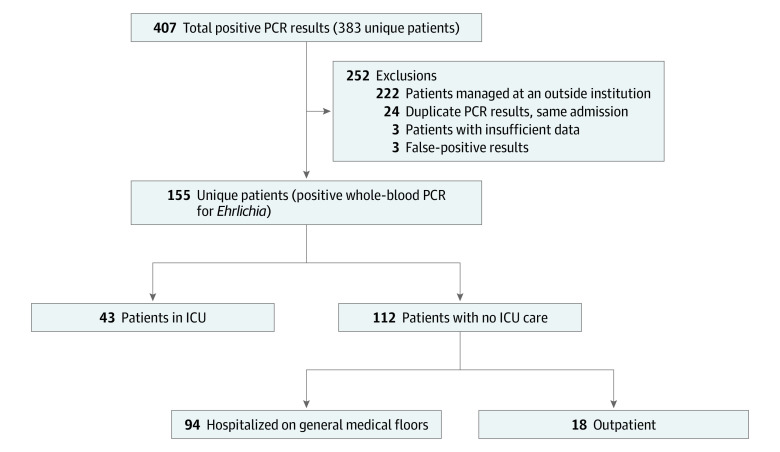
Identification of Patients With Ehrlichiosis Meeting Study Eligibility Criteria ICU indicates intensive care unit; PCR, polymerase chain reaction.

A total of 155 unique patients met study criteria, with a median age of 50 years (interquartile range, 23-64 years); 99 patients (63.9%) were men, 56 patients (36.1%) were women, and 145 patients (93.5%) identified as non-Hispanic White (Table 1). These study cases represented 40.5% of the 383 unique patients with positive PCR test results (Figure 1). Excluded cases included 222 samples submitted as reference testing from an outside institution with no clinical information, 3 cases with insufficient clinical data to evaluate severity of illness, and 3 tests deemed to be false-positive results based on the absence of a clinically compatible illness consistent with the CDC case definition.^7^

**Table 1.** Demographic and Clinical Characteristics of 155 Patients With Ehrlichiosis

Characteristic	ICU care, No. (%)		*P* value
	Yes (n = 43 [27.7%])	No (n = 112 [72.3%])	
**Demographic characteristics**			
Age, median (IQR), y	45 (15-60)	51 (27-65)	.23
Sex			
Women	19 (44.2)	37 (33.0)	.19
Men	24 (55.8)	75 (67.0)	.19
Race			
Non-Hispanic			.59
White	39 (90.7)	106 (94.6)	
Black	2 (4.7)	3 (2.7)	
Hispanic	1 (2.3)	2 (1.8)	
Asian	1 (2.3)	1 (0.9)	
**Clinical characteristics**			
Immunosuppression^a^	9 (20.9)	47 (42.0)	.02
HIV with CD4^+^ cell count, /μL			
≤200	3 (7.0)	3 (2.7)	
>200	0	10 (8.9)	
Received prednisone, ≥20 mg/d, or equivalent	0	5 (4.5)	
Organ transplant	3 (7.0)	24 (21.4)	
Baseline neutropenia	1 (2.3)	3 (2.7)	
Systemic chemotherapy	0	7 (6.3)	
Splenectomy	1 (2.3)	1 (0.9)	
Azathioprine	1 (2.3)	1 (0.9)	
Methotrexate	1 (2.3)	3 (2.7)	
Charlson Comorbidity Index level ≥4	16 (37.2)	31 (27.7)	.25
Trimethoprim plus sulfamethoxazole within the previous 2 wk	17 (39.5)	38 (33.9)	.51
Self-reported tick exposure	26 (60.5)	88 (80.7)^b^	.009

Abbreviations: ICU, intensive care unit; IQR, interquartile range.

^a^ Immunosuppression was defined as the presence of one of the following in the 3 months preceding diagnosis: HIV infection, neutropenia (neutrophil count ≤0.5 ×10^3^/μL [to convert to ×10^9^/L, multiply by 1]), end-stage kidney disease with chronic dialysis, solid organ or bone marrow transplantation, or use of immunosuppressing medications, defined as corticosteroids administered at 20 mg/d or more of oral prednisone or equivalent, tumor necrosis factor-α inhibitors, anti-CD20 monoclonal antibodies, systemic chemotherapy, or other immunosuppressant medications, such as azathioprine and methotrexate.

^b^ Data available on 109 patients.

Among study cases, the index positive test included whole blood (94.8%), cerebrospinal fluid (4.6%), and bronchoalveolar lavage fluid (0.6%). Serum obtained during the acute phase of the illness was tested for antibodies to *E chaffeensis* in 68 cases (43.9%), with 36.8% having an elevated titer (IgG ≥1:64 or IgM ≥1:16). Convalescent serologic testing was not performed in any patient.

Hospitalization was indicated in 137 cases (88.4%): 43 patients (27.7%) required ICU admission, 94 patients (60.6%) were hospitalized on general medical floors, and care for 18 patients (11.6%) was managed in the outpatient setting. Indications for ICU admission included hypotension (systolic blood pressure ≤90 mm Hg and/or mean arterial pressure ≤70 mm Hg) in 33 of the 43 patients (76.7%), with 26 of these patients (78.8%) requiring vasopressor support, need for mechanical ventilation in 17 (39.5%), and secondary hemophagocytic lymphohistiocytosis in 3 (7.0%). There were no statistically significant demographic differences between those requiring and those not requiring ICU care (Table 1).

### Clinical Findings

Overall, 56 of 155 patients (36.1%) were immunosuppressed, the median Charlson Comorbidity Index rating was 2 (IQR, 0-4), and 55 patients (35.5%) had received trimethoprim plus sulfamethoxazole in the 2 weeks before diagnosis. Causes of immunosuppression included HIV infection with CD4^+^ cell count less than or equal to 200/μL (n = 6), HIV infection with CD4^+^ cell count greater than 200/μL (n = 10), administration of prednisone or equivalent 20 mg/d or greater (n = 5), organ transplant (n = 27), neutropenia (n = 4), chemotherapy (n = 7), splenectomy (n = 2), azathioprine use (n = 2), or methotrexate use (n = 4). Those not requiring ICU care were more likely to be immunosuppressed than those requiring ICU care (42.0% vs 20.9%; *P* = .02). Data regarding self-reported tick exposure were available for 152 of 155 patients (98.1%): exposure was significantly more common in patients not requiring ICU care vs those requiring ICU care (88 of 109 [80.7%]) vs 26 of 43 [60.5%]; *P* = .009) (Table 1). Thus, overall tick exposure occurred in 114 of 155 patients (73.5%).

Signs and symptoms at presentation are reported in Table 2. A higher proportion of patients requiring ICU admission vs those not admitted to the ICU had shortness of breath (27.9% vs 9.1%; *P* = .005), altered mentation (46.5% vs 12.6%; *P* < .001), or seizures (9.3% vs 0%; *P* = .006). Rash was present in 30.2% of the ICU group compared with 16.2% of the non-ICU group (*P* = .05) but was dependent on age: the median age of patients with rash was 15 years (IQR, 11-61 years; range, 1-83 years) and that of patients without rash was 53 years (IQR, 41-65 years; range, 4-88 years) (*P* < .001).

**Table 2.** Symptomatic Presentation and Laboratory Findings of 155 Patients With Ehrlichiosis

Variable	No. of patients^a^	ICU care, No. (%)		*P* value
		Yes (n = 43 [27.7%])	No (n = 112 [72.3%])	
Fever	154	41 (95.3)	105 (93.8)	>.99
Rash	154	13 (30.2)	18 (16.1)	.05
Fatigue	153	31 (72.1)	70 (62.5)	.32
Diarrhea	154	13 (30.2)	35 (31.3)	.88
Abdominal pain	153	7 (16.3)	23 (20.5)	.65
Nausea/vomiting	154	16 (37.2)	43 (38.4)	.86
Cough	154	10 (23.3)	32 (28.6)	.54
Shortness of breath	153	12 (27.9)	10 (8.9)	.005
Myalgias	151	19 (44.2)	52 (46.4)	.92
Arthralgias	151	7 (16.3)	21 (18.8)	>.99
Headache	151	25 (58.1)	81 (72.3)	.13
Meningismus	154	2 (4.7)	8 (7.1)	.73
Seizures	154	4 (9.3)	0	.006
Altered mental status	154	20 (46.5)	14 (12.5)	<.001
Acute kidney injury	153	25 (58.1)	32 (28.6)	<.001
WBC count, median (IQR), /μL		3100 (2000-5800)	3200 (2100-4900)	.35
Nadir platelet count, median (IQR), ×10^3^/μL	154	45 (31-62)	78 (58-112)	<.001
Sodium, median (IQR), mEq/L	153	134 (121-137)	134 (131-137)	.35
AST, median (IQR), U/L	154	153 (103-419)	77 (47-147)	.004
ALT, median (IQR), U/L	154	96 (52-179)	62 (39-117)	.14
ALP, median (IQR), U/L	151	138 (75-243)	95 (65-164)	.15
Total bilirubin, median (IQR), mg/dL	150	1.6 (0.9-2.7)	0.9 (0.6-1.2)	<.001
CSF WBC, median (IQR), /μL	36	27 (10-196)	3 (1-34)	.09

Abbreviations: ALP, alkaline phosphatase; ALT, alanine aminotransferase; AST, aspartate aminotransferase; CSF, cerebrospinal fluid; ICU, intensive care unit; IQR, interquartile range; WBC, white blood cell.

SI conversion factors: To convert ALT to microkatals per liter, multiply by 0.0167; ALP to microkatals per liter, multiply by 0.0167; AST to microkatals per liter, multiply by 0.0167; bilirubin to micromoles per liter, multiply by 17.104; platelets to ×10^9^/L, multiply by 1; sodium to millimoles per liter, multiply by 1; WBC to ×10^9^/L, multiply by 0.001).

^a^ Values indicate number of patients with data available.

### Laboratory Findings

Patients requiring ICU care vs those not admitted to the ICU were more likely to have acute kidney injury (58.1% vs 29.1%), thrombocytopenia (median platelet count, 45 × 10^3^/μL vs 78 × 10^3^/μL [to convert to ×10^9^/L, multiply by 1]), higher aspartate aminotransferase levels (median, 153 U/L vs 77 U/mL [to convert to microkatals per liter, multiply by 0.0167]), and higher bilirubin levels (median, 1.6 mg/dL vs 0.9 mg/dL [to convert to micromoles per liter, multiply by 17.104]). There was no significant difference in the median white blood cell count at presentation between the ICU (3100/μL) vs the no ICU (3200/μL) groups (Table 2).

### Treatment and Outcomes

**Figure 2.**
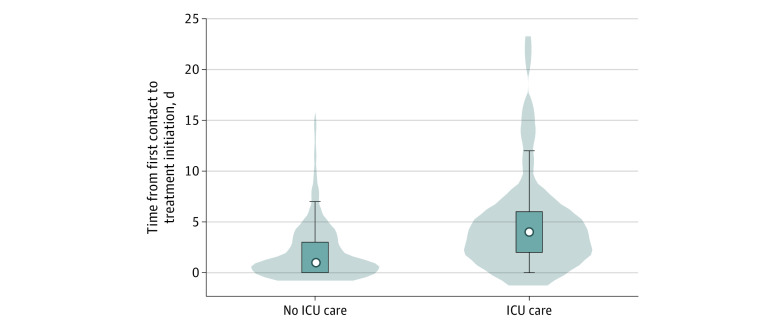
Distribution of Days From First Health Care Contact to Treatment Initiation ICU indicates intensive care unit; white dots, median value; vertical bars, interquartile range; and center vertical line, range (excluding outliers).

All patients with ehrlichiosis were treated with doxycycline. Patients requiring ICU care had a significantly longer delay from onset of symptoms to diagnostic testing (median, 7 vs 6 days; *P* = .002) and doxycycline initiation (median, 7 vs 5 days; *P* = .008). Patients admitted to the ICU also had a longer delay from initial interaction with the health care system to diagnostic testing (median, 4 vs 1 day; *P* < .001) and doxycycline initiation (median, 4 vs 1 day; *P* < .001) (Figure 2). There was no significant difference in median time from diagnostic testing to treatment initiation between ICU and non-ICU patients (0 vs 0 days; *P* = 1.0). Median time to doxycycline initiation from symptom onset did not differ significantly by reported tick exposure (tick exposure compared with those who did not report tick exposure, 6 days [IQR, 4-7] vs 6 days [IQR, 4-8]; *P* = .96).

Case fatality rates were 3.9% overall (6 of 155 patients: 5 required ICU care and 1 was admitted to a regular inpatient unit) and 11.6% among patients requiring ICU care (5 of 43 patients). Length of stay exceeded 7 days for 31.4% of hospitalized patients. The median length of stay for patients requiring ICU care was 6 days (IQR, 4-14 days) compared with hospitalized patients who did not require ICU stay (4 days [IQR, 3-6 days], *P* = .001).

In adjusted analysis, only the median time from initial health care interaction to initiation of doxycycline therapy was independently associated with an increased risk for ICU care (aPR, 1.09; 95% CI, 1.04-1.14; *P* < .001) (Table 3). Conversely, there appeared to be a nonsignificant change toward a decreased need for ICU care among immunosuppressed persons (aPR, 0.51; 95% CI, 0.26-1.00; *P* = .05). Documentation of tick exposure was independently associated with a decreased need for ICU care (aPR, 0.54; 95% CI, 0.34-0.86; *P* = .01).

**Table 3.** Unadjusted and Adjusted Prevalence Ratio of Intensive Care Unit Care Among Patients With Ehrlichiosis

Variable	Prevalence ratio (95% CI)	
	Unadjusted	Adjusted
Age, y		
<10	1.51 (0.83-2.74)	0.84 (0.39-1.81)
10-20	1.38 (0.83 -2.32)	0.90 (0.50-1.61)
21-30	1.26 (0.73-2.18)	0.95 (0.54-1.67)
31-40	1.13 (0.75-1.71)	0.99 (0.65-1.51)
41-50	1 [Reference]	1 [Reference]
51-60	0.95 (0.68-1.31)	0.97 (0.70-1.34)
61-70	1.01 (0.61-1.68)	0.89 (0.54-1.44)
>70	1.16 (0.42 -3.18)	0.79 (0.30 -2.13)
Women	1.40 (0.84-2.32)	1.32 (0.74-2.37)
Race		
Non-Hispanic White	1.49 (0.66-3.34)	1.66 (0.81-3.41)
Immunosuppression	0.47 (0.24-0.91)	0.51 (0.26-1.00)
Trimethoprim plus sulfamethoxazole exposure	1.19 (0.71-1.99)	1.36 (0.77-2.38)
Reported tick exposure	0.55 (0.33-0.90)	0.54 (0.34-0.86)
Rash	1.72 (1.02-2.89)	1.74 (0.92-3.30)
Time from first contact to treatment initiation (per day)	1.09 (1.06-1.13)	1.09 (1.04-1.14)

## Discussion

We identified 155 unique patients with laboratory-confirmed ehrlichiosis between 2007 and 2017. Of these, 137 patients (88.4%) were hospitalized, 43 patients (27.7%) required ICU care, and 6 patients (3.9%) died during the index hospitalization. In contrast, of the 3593 cases reported to the CDC between 2008 and 2012, only 57% required hospitalization and 11% had a life-threatening complication.^7^ Several factors limit the ability to compare these data directly. In contrast to the present study with all cases confirmed by PCR and which would be categorized as confirmed ehrlichiosis, more than two-thirds of the cases identified through CDC National Notifiable Disease Surveillance were classified as probable infections. The finding of less severe disease in the surveillance population may reflect a bias introduced by including probable cases, classified as patients with a single elevated titer, which may reflect false-positive diagnoses due to prior exposure or cross-reactive antibodies. Alternatively, despite the high analytic sensitivity of PCR to detect ehrlichial DNA, estimated between 52% and 87%,^29,30,31^ there is the potential for mild cases with low bacteremic burden to provide negative test results.^32^ Taking these considerations into account, the hospitalization (88.4%) and case fatality (3.9%) rates in our study were higher than the 77% hospitalization and 2% case fatality rates among the more analogous subset of confirmed cases reported to the CDC.^7^

A commonly cited risk factor for severe disease is immunosuppression.^25^ A review from 2002 reported a 25% mortality among immunocompromised patients.^14^ More recently, national surveillance data found an increased risk of hospitalization (relative risk [RR], 1.4), life-threatening complications (RR, 2.4), or death (RR, 2.3) among immunocompromised patients.^7^ A study of 25 patients with ehrlichiosis who had undergone transplantation found a significantly higher rate of ICU care among lung transplant recipients compared with other solid organ transplants.^11^

In our population there was an unexpected change (*P* = .05) for immunocompromising conditions to be protective against ICU admission. This possible association held true even when controlling for potential confounders, such as use of trimethoprim plus sulfamethoxazole and time to initiation of doxycycline therapy. This finding may reflect a higher proportion of less virulent *E ewingii* infections in immunocompromised patients.^22,23,33^ As the assay used in the present study was unable to differentiate between *Ehrlichia* species, we were unable to confirm this hypothesis. Alternatively, the decreased severity of infection in the immunocompromised patients in our study may have been due to a higher linkage to care and prompt medical evaluation. Because the case definition for immunocompromise in our study excluded diabetes, our findings cannot be directly compared with the national surveillance data, which included diabetes as an immunocompromising state.

Other factors previously associated with increased severity of ehrlichiosis were not identified in our study. In national surveillance data, the case fatality rate for ehrlichiosis was significantly increased in children younger than 5 years (4%) and older individuals (>70 years) (3%) compared with the overall case fatality rate of 1%.^7^ However, age did not appear to be associated with increased risk of ICU care in our study, although the numbers of children aged 5 years or younger (n = 6) and patients aged 65 years or older (n = 35) were low. Prior treatment with trimethoprim plus sulfamethoxazole has been associated with severe or fatal disease in a number of anecdotal case reports/series.^15,16,17,18,19,20,21,22^ Controlling for potential confounders, such as comorbid disease and delay in doxycycline therapy initiation, we did not find antecedent use of trimethoprim plus sulfamethoxazole to be correlated with disease severity.

In the present study, only 2 factors were statistically significantly associated with the need for ICU care in adjusted analysis: recent tick exposure and delay in doxycycline therapy initiation. A documented history of tick exposure was present in 73.5% of patients overall but was significantly less common in the group requiring ICU care (aPR, 0.54; 95% CI, 0.34-0.86). Absence of tick bite has been associated with delayed diagnosis and increased mortality among other tickborne infections,^34^ presumably due to failure to consider the diagnosis and initiate empirical therapy with doxycycline. However, the inverse association between documented tick exposure and need for ICU care held true in the present study even after controlling for the timeliness of doxycycline therapy. We hypothesize that this association may reflect that sicker patients had cognitive impairment leading to limitations in recall or communication of tick exposure. Therefore, lack of documented tick exposure should not dissuade clinicians from a diagnosis of ehrlichiosis.

The second variable independently associated with severe ehrlichiosis appears to be a delay in time from initial health care encounter to doxycycline therapy initiation. The importance of early antibiotic treatment has been well established for spotted fever rickettsioses with delay in therapy associated with severe disease, morbidity, and death.^34,35,36,37^ This association has been less clearly proven for ehrlichiosis. An early study reported that initiation of doxycycline within 5 days of symptom onset was associated with better outcomes.^38^ A more recent case series noted that patients who had more than a 24-hour delay in initiation of doxycycline therapy after hospital admission were more likely to need ICU care (39% vs 0%) and had longer hospitalization (12 vs 4 days) and duration of illness (21 vs 9 days).^39^

Historically, there has been concern among pediatricians regarding prescription of doxycycline in children owing to the perceived risk of dental discoloration. In a survey of more than 1500 health care clinicians, only 35% chose doxycycline to treat Rocky Mountain spotted fever in children younger than 8 years.^40^ More recent studies have refuted the association between short-course doxycycline therapy and tooth discoloration^41^ and empirical use of doxycycline is now strongly recommended by the CDC and American Academy of Pediatrics for all age groups with suspected rickettsial infection.^42^ Our study noted that patients with more severe ehrlichiosis had a statistically significant delay from initial health care interaction to initiation of doxycycline therapy, supporting the importance of early initiation of doxycycline treatment irrespective of age.

### Strengths and Limitations

To our knowledge, this study is the first to include a large number of patients, both hospitalized and nonhospitalized patients, and definitive laboratory confirmation of acute infection through PCR testing. The use of rigorous molecular diagnostic testing sets this study apart from older studies that relied on serologic diagnosis.

Several limitations should be considered in interpreting the findings of this observational study. It is likely that this study was biased toward inclusion of more severely ill patients, as mild illness may be treated empirically without laboratory confirmation or may be missed by PCR if the bacterial burden is low. Similarly, the study population was accrued from a single tertiary care facility, and it is likely that the population was biased toward more severe cases who were transferred for a higher level of care. In addition, the *Ehrlichia* PCR test used by the clinical laboratory does not distinguish between the 3 species of *Ehrlichia* and between *Ehrlichia* and *Anaplasma*, a closely related tick-borne pathogen. In the study catchment area, only 2 species—*E chaffeensis and E ewingii*—are endemic.^43^ *Ehrlichia ewingii* has been shown both to be more common in immunocompromised hosts and to cause milder disease.^22,23,33^

## Conclusions

Reported cases of ehrlichiosis have increased by more than 70% since implementation of the current surveillance case definition adopted in 2008.^7^ The elucidation of modifiable risk factors for severe disease is critical to improving outcomes. Coronavirus disease 2019, which may present in a similar fashion, adds further relevance to the early diagnosis and management of ehrlichiosis. Delay in initiation of doxycycline therapy despite interaction with a health care clinician remains a significant factor associated with severe disease and underscores that knowledge gaps in the treatment of tickborne infections persist.^40,44^ Clinician education may be a key factor in improving outcomes of this increasingly common infection.
